# Quantitative Simulations of Siloxane Adsorption in Metal–Organic Frameworks

**DOI:** 10.1021/acsami.3c07158

**Published:** 2023-07-26

**Authors:** Jia Yuan Chng, David S. Sholl

**Affiliations:** † School of Chemical & Biomolecular Engineering, Georgia Institute of Technology, Atlanta, Georgia 30332-0100, United States; ‡ Oak Ridge National Laboratory, Oak Ridge, Tennessee 37830, United States

**Keywords:** force fields, molecular simulation, adsorption, metal−organic frameworks, siloxanes

## Abstract

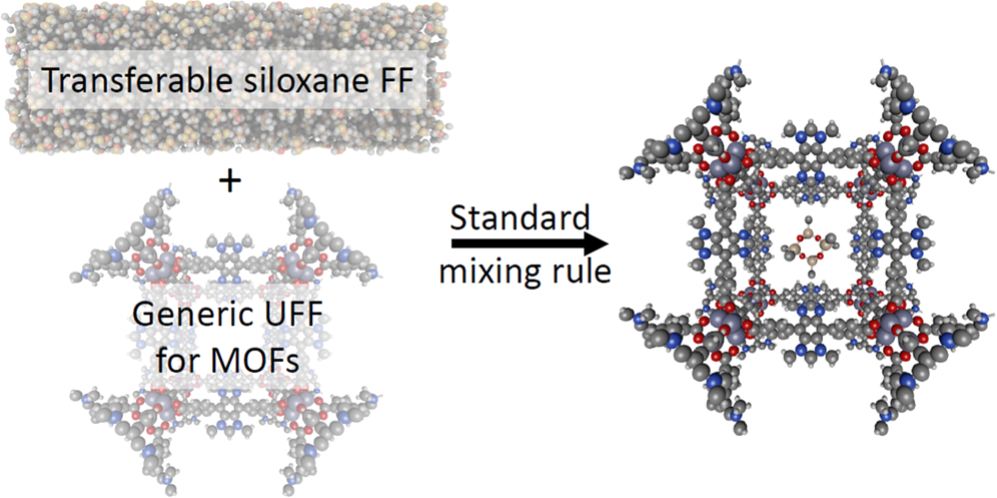

We present a transferable force field (FF) for simulating the bulk properties of linear and cyclic siloxanes and the adsorption of these species in metal–organic frameworks (MOFs). Unlike previous FFs for siloxanes, our FF accurately reproduces the vapor–liquid equilibria of each species in the bulk phase. The quality of our FF combined with the Universal Force Field using standard Lorentz–Berthelot combining rules for MOF atoms was assessed in a wide range of MOFs without open metal sites, showing good agreement with dispersion-corrected density functional theory calculations. Predictions with this FF show good agreement with the limited experimental data for siloxane adsorption in MOFs that is available. As an example of using the FF to predict adsorption properties in MOFs, we present simulations examining entropy effects in binary linear and cyclic siloxane mixture coadsorption in the large-pore MOF with structure code FOTNIN.

## 1 Introduction

The low surface tension, low water solubility, high thermal stability, and low toxicity of siloxanes have led to widespread uses in consumer, healthcare, and industrial products. Some examples include the manufacture of cosmetics, paints, mechanical fluids, and rubber. The presence of siloxanes in the environment is therefore unsurprising.^1^ Linear and cyclic siloxanes are abbreviated by “L” and “D”, respectively, and the number that follows represents the number of silicon atoms in the siloxane. For example, D4 is octamethylcyclosiloxane and L4 is decamethyltetrasiloxane. The degradation of silicone polymers in landfills or wastewater treatment plants produces volatile methylsiloxane byproducts such as L2 and D4, with D4 making up about 70% of the total siloxane contaminants in biogas.^2−4^ Siloxanes are known to cause fouling of biogas capture equipment and catalysts, hampering the effective use of biogas.

Among various separation technologies proposed to treat D4 in biogas,^5−7^ adsorption technology using activated carbon as the adsorbent is the only one used industrially.^2^ However, in most cases, activated carbons in this application are not readily regenerable by physical or chemical means. Silica gel^5,8^ and more recently metal–organic frameworks (MOFs)^6,9,10^ have been shown to be regenerable sorbents for siloxanes when heated. Computational and experimental studies of MOFs by Gulcay-Ozcan et al. showed that the large-pore MOF FOTNIN outperforms DUT-4^9^ and MIL-101^10^ in both adsorption capacity and regenerability under mild conditions.^11^

Metal–organic frameworks (MOFs) are a diverse class of porous crystalline solids formed by the coordination of metal ions with organic linkers. MOFs have been widely studied for various separation, catalysis, and gas storage applications.^12^ Because tens of thousands of distinct MOFs exist,^13−15^ screening of MOFs as adsorbents using molecular simulations has become an important complement to direct experimental searches for high-performance materials.^16−21^ The success of molecular simulation in MOFs relies on the availability of accurate force fields (FFs) for adsorbate–MOF and adsorbate–adsorbate interactions.^22^ FFs for adsorbate–adsorbate interactions are typically developed to reproduce experimental vapor–liquid equilibrium curves. Several FFs have been developed for poly(dimethylsiloxane) (PDMS) to describe the thermodynamic and structural properties of pure PDMS melts^23−28^ and the prediction of solubility coefficients of light gases and hydrocarbons in PDMS.^29^ However, no similar FF has been developed for volatile linear methylsiloxanes. Gulcay-Ozcan et al. used nonbonded parameters from the generic Universal Force Field (UFF) for all atoms in D4 in their work on siloxane adsorption.^11^ An alternative FF for siloxane interactions was introduced by Matsubara et al., who developed a Lennard-Jones-type potential for D4 in the solid and liquid phases.^30^ We show below that both of these FFs make poor predictions of the liquid phase densities for short-chain siloxanes (Figure S1). Since adsorption in nanoporous materials often creates a local environment with liquid-like molecular densities, using FFs that make accurate predictions for these densities is likely to be important for accurately simulating adsorption.

In addition to an accurate FF for adsorbate–adsorbate interactions, quantitative simulation of adsorption in MOFs requires the use of accurate adsorbate-framework FFs. The work of Gulcay-Ozcan et al.^11^ used a widely adopted set of mixing rules to define a siloxane–MOF FF based on the UFF but did not assess the accuracy of this approach. A useful strategy for examining the accuracy of adsorbate-framework FFs is to compare the adsorption energies computed with an FF with adsorption energies computed with dispersion-corrected density functional theory (DFT) calculations.^31−33^ No comparison of this kind has been made to date for siloxane adsorption in MOFs or other nanoporous adsorbents.

In this paper, we report a new transferable molecular force field for cyclic and linear siloxanes for which VLE data are available from the NIST Webbook.^34^ Specifically, we introduce a united-atom (UA) FF for D4, D5, D6, L2, L3, L4, L5, and L6 that makes predictions in good agreement with bulk VLE data. We define a transferable FF for the adsorption of these molecules in MOFs by combining our new adsorbate–adsorbate FF with MOF interaction parameters from UFF and make an extensive comparison of adsorption energies from this FF with DFT binding energies for D4 in 55 non-open metal site MOFs. This extensive data set indicates that our new FFs are suitable for making quantitative predictions of siloxane adsorption in MOFs. D4 adsorption isotherms in FOTNIN and MIL-101 using this force field also captures the pattern of the experimental measurements from Gulcay-Ozcan et al.^11^ Having demonstrated the accuracy and transferability of this FF, we illustrate its use for predicting adsorption properties that would be challenging to determine experimentally by examining the separation of binary mixtures of linear and cyclic siloxanes (L2/D4, L4/D4, and L4/D5) in FOTNIN.

## 2 Transferable Force Fields for Cyclic and Linear Siloxanes

### 2.1 Model

Siloxane consists of a silicon–oxygen backbone, with two methyl groups attached to each silicon atom. The UA model treats each methyl group as a single interacting site, and each Si and O atom as an interacting site. We investigated FFs in which nonbonded interactions between pseudoatoms interact via a Lennard-Jones potential
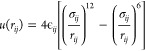
1where *r*_*ij*_ is the separation between pseudoatoms, ϵ_*ij*_ is the LJ potential well depth, and σ_*ij*_ is the LJ segment diameter. Lennard-Jones interactions are truncated at 14 Å, and analytical tail corrections are applied. Intramolecular 1–4 interactions are excluded in this force field.

Partial charges for Si, O, and CH_3_ in D4 were computed using the density-derived electrostatic and chemical (DDEC6) method^35^ at the PBE-D3 DFT level of theory. The Coulombic interaction in a D4–D4 dimer was compared against the overall intermolecular interaction energy computed with functional symmetry-adapted perturbation theory with D3 dispersion corrections and the refitted modified Becke–Johnson damping function (F-SAPT0-D3M(BJ)) using the jun-cc-pVDZ basis set.^36^Figure S2 shows the contribution of Coulombic interactions between two D4 molecules is negligible. On this basis, Coulombic interactions are neglected in our siloxane FF.

To develop intramolecular parameters for the FF, geometry optimization for all siloxane molecules was performed at the second-order Møller–Plesset perturbation (MP2) level of theory using the 6-311G(d,p) basis set. Optimized bond lengths were then averaged and kept fixed as summarized in Table S1. Bond bending within molecules was modeled by a simple harmonic potential
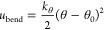
2where θ, θ_0_, and *k*_θ_ are the bending angle, MP2/6-311G(d,p) optimized bending angle, and force constant, respectively. θ_0_’s are calculated by averaging across the bending angles of the optimized siloxane structures. Force constants for cyclic siloxanes were adopted from the TraPPE force field for oxanes,^37,38^ while values for linear siloxanes were taken from a previous poly(dimethylsiloxane) force field.^28^ Bond bending parameters are summarized in Table S2.

Torsional flexibility for cyclic siloxanes was represented by the OPLS torsional potential^39^
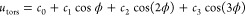
3Torsional potentials of linear siloxanes have the following form
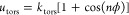
4Torsional parameters for cyclic and linear siloxanes were taken from the TraPPE force field for oxanes^37,38^ and a united-atom force field for PDMS, respectively.^28^ Torsional parameters are summarized in Table S3.

Validation of the siloxane FF was performed by comparison with pure species siloxane VLE data available in the NIST Webbook.^34^ Because adsorption in nanopores is likely to create liquid-like densities, we focus on our FF’s description of the liquid densities.

### 2.2 Computational Methods

We used molecular dynamics (MD) to perform molecular simulations of pure siloxane fluids using LAMMPS.^40^ MD and Gibbs Ensemble Monte Carlo (GEMC) can both be applied to calculate vapor–liquid equilibrium properties.^41^ The MD approach has been used extensively in recent years to perform VLE simulations of water,^42^ various light gases,^43^ and hydrocarbons.^44,45^ A limitation to using MD to simulate coexisting phases is that for systems with high interfacial free energies, long equilibration times may be needed.^46^ Test calculations were conducted to compute the VLE of methane for the TraPPE force field^47^ using MD and GEMC. The MD method gives good agreement with results from GEMC,^47^ exemplifying the validity of this MD approach (Figure S4).

MD simulations were performed in the canonical ensemble (*NVT*), with *N* molecules placed in a parallelepiped simulation box of constant volume *V* at a fixed temperature *T*. Initialization of the simulations followed the recommendations of Muller et al.^41^ In all cases, the rectangular parallelepiped has dimensions *L*_*x*_ = 65 Å × *L*_*y*_ = 65 Å × *L*_*z*_ = 260 Å(= 4*L*_*x*_), which typically contained ∼19,000 atoms. Temperature was regulated using a Nosé–Hoover thermostat with a relaxation constant of 1.0 ps.^48^ MD simulations were carried out with a time step of 1 fs.

The initial configuration was first simulated at a temperature above its critical state for at least 5 ns in order to homogenize the system (Figure S5a).^34^ The temperature of the system was then quenched to the desired value and equilibrated for at least 10 ns bbefore statistics were collected (Figure S5b). The vapor and liquid phase densities were obtained by ignoring the regions associated with the vapor–liquid interface and averaging across the gas and liquid regions only, with the boundaries of these regions being identified by visual inspection.^49^

We started the FF fitting for cyclic siloxanes to their vapor–liquid coexistence curves (VLCC) by adjusting the LJ parameters for O and CH_3_ in a stepwise manner. Considering the steric shielding of silicon atoms in siloxanes, the ϵ parameter for Si was set to zero. For consistency and simplicity, σ for Si was included by adopting the value from a previous poly(dimethylsiloxane) force field.^28^ Initial estimates for σ and ϵ for O and CH_3_ were taken from the TraPPE-UA model for ethers^38^ and oxanes.^37^ The fitting process was an iterative one. Because the initial estimate for the σ parameters from TraPPE-UA turned out to be appropriate, major adjustments were made to the CH_3_ and O’s ϵ parameters. Specifically, these parameters were increased to stretch the phase envelope closer to the critical points. The σ parameter for CH_3_ was then increased slightly to reduce the saturated liquid densities. The finalized parameters are summarized in Table 1.

**Table 1:** Lennard-Jones Parameters for Siloxanes

pseudoatoms	σ (Å)	ε (kcal/mol)
Si	3.385	0.0000
O	2.390	0.1520
CH3	3.850	0.3776

**Figure 1:**
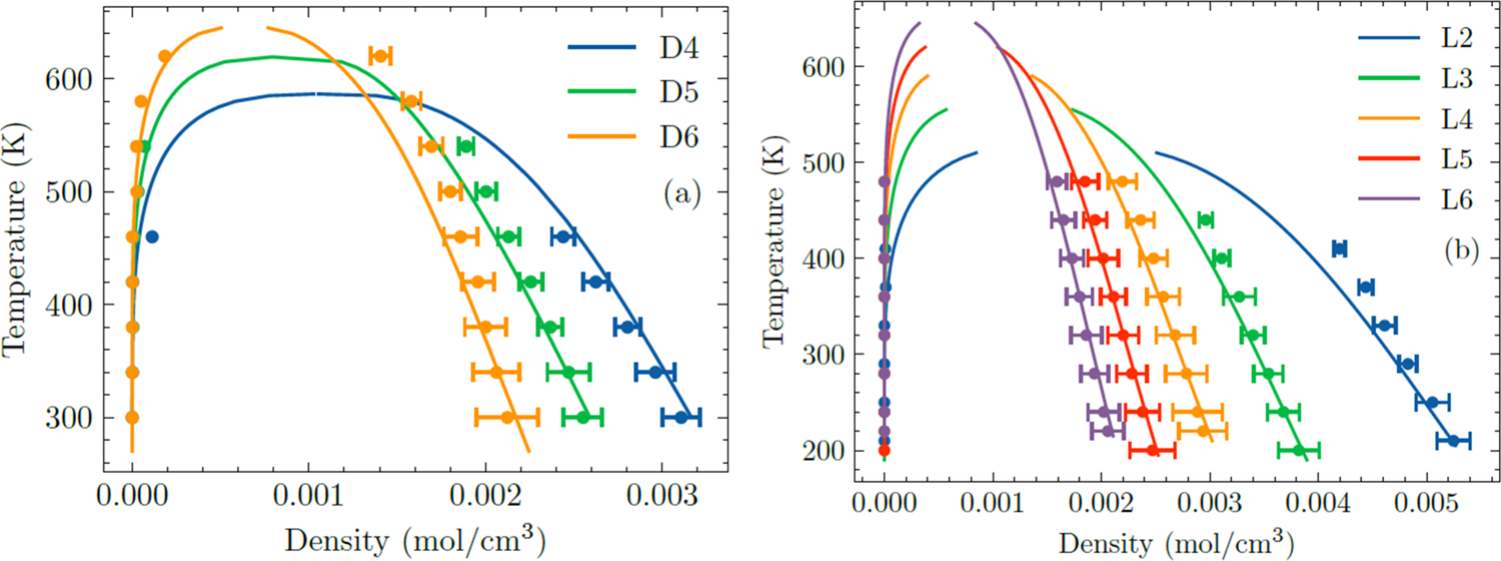
Vapor–liquid coexistence curves for (a) cyclic and (b) linear siloxanes, with molecular simulation data shown as dots and experimental data from the NIST Webbook shown as solid curves. The VLE curves in (a) were used during the fitting of the FF, while the VLE curves in (b) were not.

The LJ parameters fitted for D4, D5, and D6 were used for the linear L2–L6 siloxanes without further adjustments. The vapor–liquid coexistence curves for D4–D6 fitted by our FF and the FF’s predictions for L2–L6, along with data from the NIST Webbook^34^ are shown in Figure 1. The saturated liquid densities for both sets of molecules are in far better agreement with experimental data than the earlier FFs shown above. A slight overestimation of the density at higher temperatures is observed since these LJ parameters were not fitted to reproduce the critical temperatures. Nevertheless, our results indicate that the performance of this FF for molecular simulations well below the critical temperatures of the molecules is satisfactory. Although our focus below is on using these FFs as part of simulations of siloxane adsorption in MOFs, this is the first time that an FF suitable for the bulk properties of siloxanes has been reported, so we anticipate that this FF will also be useful in other settings.

## 3 Force Field for D4–MOF Interactions

Having developed accurate FFs for siloxane–siloxane interactions, we now turn to describing the interactions between siloxanes and MOFs. To this end, we focus on the binding energies of a representative siloxane species, D4, in a wide variety of MOFs. Our aim is to develop an FF for siloxane–MOF interactions that makes predictions consistent with the binding energies from dispersion-corrected DFT calculations, specifically, PBE-D3 calculations. Force fields for adsorbate–adsorbent interactions derived with reference to first-principles approaches have been shown to accurately predict the adsorption of various adsorbates in MOFs^50,51^ and zeolites.^22,52^

To avoid biasing our assessment by focusing on a single MOF, we made comparisons for a wide range of potential adsorbents. Specifically, we selected the 10 top performing MOFs for D4 capture as ranked by Gulcay-Ozcan et al.^11^ and the 45 non-open metal sites MOFs with the largest pore volume from the 2019 Computation-Ready, Experimental All Solvent Removed (CoRE ASR) MOF database.^14^ Since 6 out of these 45 MOFs were identified by Gulcay-Ozcan et al., the subsequent 6 non-open metal sites MOFs with large pore volumes were chosen from the CoRE MOF Database. This gives a total of 55 non-open metal site MOFs for which we made comparisons. These materials and some of their characteristics are listed in Table S4. MOF structures were taken from the CoRE MOF database and used without relaxation or further adjustment. We compared results for two different siloxane–MOF FFs, one based on the siloxane FF we introduced above and another using the UFF-based siloxane potential from Gulcay-Ozcan et al.^11^ In both cases, interaction parameters between siloxane groups and MOF atoms were defined by using UFF parameters for the MOF atoms and Lorentz–Berthelot combination rules.

In each MOF we generated 50 independent D4 configurations using NVT Monte Carlo (MC) at 400 K with our new siloxane force field. All D4–MOF and D4–D4 interactions are truncated at 12.8 Å, with analytical tail corrections applied for interactions beyond this cutoff. To satisfy the minimum image convention with respect to the cutoff distance, simulation cells of the adsorbents were expanded when needed such that the perpendicular lengths are at least 26 Å. MOFs were assumed to be rigid during the simulations. These configurations (MOF + one D4 molecule) were then used for single-point interaction energy calculations with the PBE-D3 DFT method in the Vienna Ab initio Simulation Package (VASP).^53,54^ All single-point DFT calculations sampled reciprocal space at the Γ point and used an energy cutoff of 400 eV.

**Figure 2:**
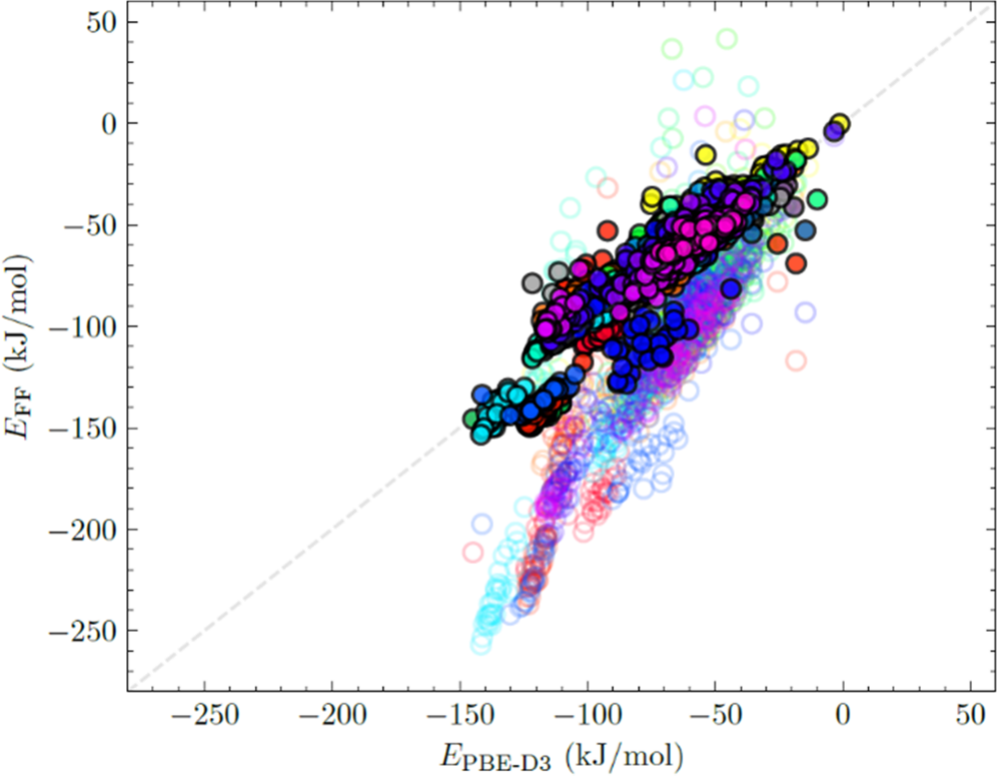
Comparison of the binding energies of D4 in 55 MOFs at the PBE-D3 level and our new FF (filled circles) and Gulcay-Ozcan et al.’s FF^14^ (unfilled circles), with data from 50 independent configurations in each MOF. MOFs are represented by different colors.

Figure 2 compares the binding energies of D4 in 55 MOFs from PBE-D3 DFT and the two siloxane–MOF FFs. A histogram of the difference between the DFT and FF energies is shown in Figure S6. Compared to PBE-D3, the FF of Gulcay-Ozcan et al. overpredicts binding energies with a mean absolute error (MAE) of 42 kJ/mol. The overestimation of the binding energies is particularly large for the most favorable states, a situation that would lead to a strong overestimation of the adsorption of D4 in MOFs at low loadings. The interaction energies calculated with our newly developed FF show significantly better agreement with those of PBE-D3, with an MAE of 7 kJ/mol. Similar comparison of the binding energies of D5, D6, L2, L3, and L4 in 5 randomly selected MOFs from the 55 MOFs also shows better agreement between our new FF and PBE-D3 (see Figure S7). These observations suggest that our new FF is suitable to describe MOF/siloxane interactions without the need for further parameter fitting to reproduce the observed DFT binding energies.

## 4 Comparisons between FF Predictions and Experimental Adsorption Isotherms

In addition to detailed comparisons with DFT data, it is useful to compare predictions from our new FF with the limited experimental data that are available for D4 adsorption in MOFs. Our new FF was used to calculate adsorption isotherms in the only two MOFs for which experimental adsorption data for D4 is available, FOTNIN^11^ and MIL-101.^55^ Simulations were performed using Continuous Fractional Component Monte Carlo (CFCMC) with the RASPA simulation package.^56^ These simulations were carried out using 200,000 MC steps, and preliminary tests indicated that this choice gave reasonable convergence. The MOF was assumed to be rigid during these simulations. The Universal Force Field (UFF) is used to model the framework atoms in MOFs. The Lorentz–Berthelot combining rules are used to compute the unlike interatomic parameters. D4–MOF interactions were truncated at 12.8 Å. FOTNIN and MIL-101 structures were taken from the CoRE MOF database^14^ and Cambridge Structural Database (CSD) MOF database,^57^ respectively, and used without relaxation or further adjustment. Pore limiting diameters (PLD), surface area, density, and helium void fraction of these materials were calculated using Zeo++^58^ and are summarized in Table 2.

**Table 2:** Properties of Defect-Free FOTNIN and MIL-101 Structures Calculated Using Zeo++^58^

MOF	PLD (Å)	surface area (m^2^/g)	density (g/cm^3^)	helium void fraction
FOTNIN	28.3	2990	0.27	0.90
MIL-101	13.9	3547	0.44	0.76

**Figure 3:**
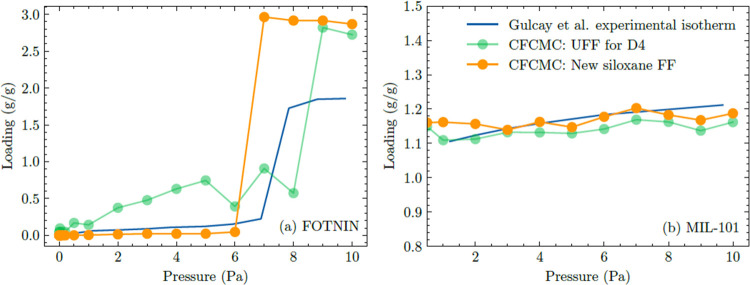
D4 simulated isotherms in (a) FOTNIN and (b) MIL-101 at 303 K using the FF of Gulcay-Ozcan et al. and our new FF compared to experimental data from Gulcay-Ozcan et al.^11^

FOTNIN is a hydrophobic MOF with closed-metal sites that was reported as a possible D4 adsorbent with higher uptake (1.8 g/g) and regenerability^11^ than the hydrophilic open metal site MOF MIL-101 (0.95 g/g).^55^ To date, only one experimental adsorption isotherm for each of FOTNIN and MIL-101 exists, as reported by Gulcay-Ozcan et al.^11^ These isotherms are shown in Figure 3. Under these conditions, the saturation pressure for D4 is 191 Pa.^34^ In both MOFs, the isotherms reach near-saturation loading at pressures lower than this saturation pressure.

We first discuss the adsorption of D4 in FOTNIN. Gulcay-Ozcan et al. did not report simulation data for this material, so we cannot compare these to previous simulation results. Figure 3a shows that our FF-predicted isotherm is in reasonable agreement with the experimental results by Gulcay-Ozcan et al. although it overpredicts the saturation capacity. Similar simulations were performed with the FF introduced by Gulcay-Ozcan et al., however, overestimate the D4 uptake in the lower pressure range and does not predict the shape of the experimentally observed isotherm as well. The difference between the experimental and simulated saturation loadings appears to have arisen because of incomplete evacuation of pores during the activation procedures used experimentally, as can be inferred from comparing the experimentally reported pore volume (2.2 cm^3^/g) and the theoretical pore volume (3.3 cm^3^/g).^11^ If the experimental data is scaled by the ratio of the theoretical and observed pore volume, the experimental isotherm and the predictions from simulations with our FF are in good agreement.

We now turn to the adsorption isotherm for D4 in MIL-101. The lowest pressure point available from the experimental data is at 1 Pa. From Figure 3b, our new FF gives reasonable agreement with the experimental adsorption isotherm. The FF of Gulcay-Ozcan et al. slightly underestimates the adsorption of D4 relative to the experiment, but the differences between the simulation results and the two FFs are not large. Unlike the case for FOTNIN, there is no indication that a pore volume correction is needed for MIL-101. Gargiulo et al. reported a D4 saturation uptake of 0.95 g/g at 298 K,^10^ but Gulcay-Ozcan et al. found the experimental and simulation D4 capacity to be 1.15 and 1.03 g/g, respectively.^11^ Although our comparison with experimental data is limited by the lack of available experimental data, the combination of our comparisons with DFT results and with the extant experimental data indicates that the FF we have introduced for D4 adsorption in MOFs makes accurate predictions. Moreover, it is reasonable to expect that the FF is fully transferable among siloxanes, meaning that our approach for the first time provides an FF that can be used to make quantitative predictions about the adsorption of a range of siloxanes in MOFs.

## 5 Adsorption of Binary Mixtures of Linear and Cyclic Siloxanes in FOTNIN

**Figure 4:**
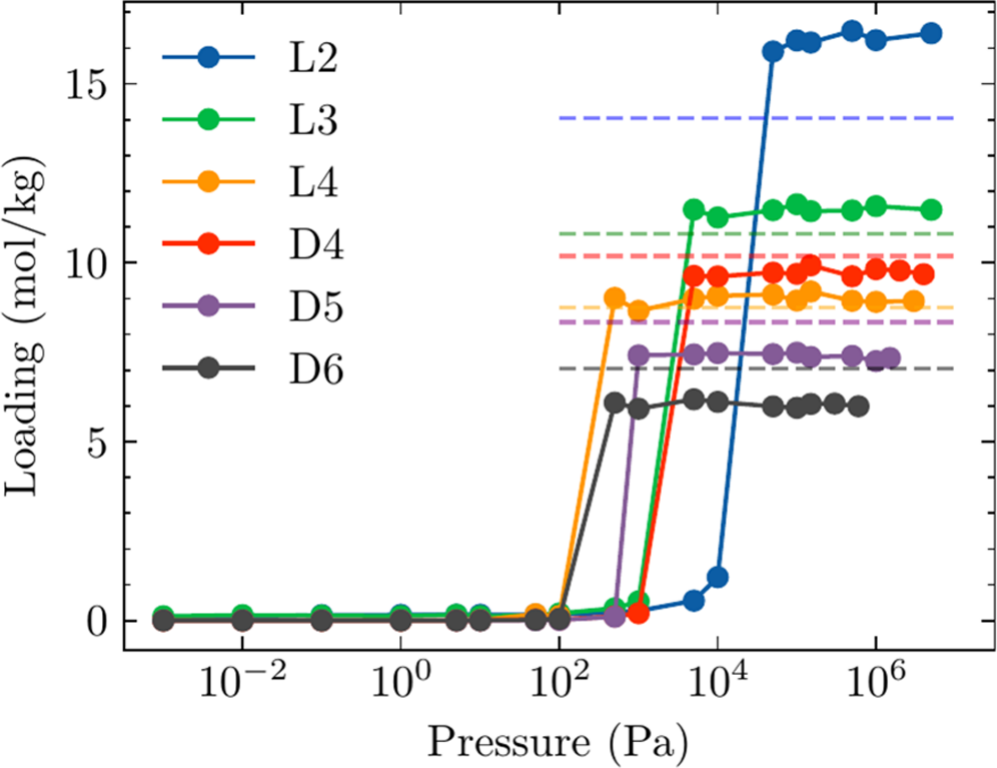
CBMC data for single-component adsorption isotherms of L2, L3, and L4, and CFCMC data for single-component adsorption isotherms of D4, D5, and D6 in FOTNIN at 435 K. Dashed lines represent the saturated loadings of siloxanes in FOTNIN calculated by combining the accessible volume of FOTNIN (0.00383 m^3^/kg_MOF_)^14^ with the siloxane’s bulk-phase liquid density (see Table S6).^34^

**Figure 5:**
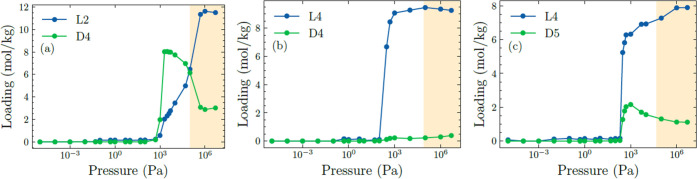
(a) CFCMC simulation of equimolar binary L2/D4 mixture adsorption in FOTNIN at 435 K. Phase transition from vapor to liquid occurs at 100,000 Pa. (b, c) CFCMC/MD simulations of equimolar binary L4/D4 and L4/D5 mixture adsorption in FOTNIN at 435 K. Phase transition from vapor to liquid occurs at 80,000 Pa for L4/D4 mixture and 50,000 Pa for L4/D5 mixture. The shaded regions indicate liquid phase operation; the transition from vapor to liquid bulk phase is determined by the Peng–Robinson equation of state.^56^

No information about the adsorption selectivity for mixtures of siloxanes in MOFs is currently available, even though any practical application of adsorption for these species is likely to involve a mixture of species. The experimental data above show that at pressures similar to the saturation pressure for a siloxane species it is reasonable to expect that the pores of MOFs with large pore volumes are almost saturated with siloxane molecules (at equilibrium). For single-component adsorption, this suggests that the pure fluid molar volume can give a reasonable estimate for the adsorbed loading under these conditions.^59^ This observation underscores the importance of using FFs that accurately represent the pure fluid liquid phase density of siloxanes (see Figures 4 and 5).

The separation of linear or cyclic siloxanes during adsorption can be achieved by the preferential adsorption of one species relative to others in an adsorbing mixture. In dense adsorbed states, it is likely that adsorption selectivity is primarily driven by entropic effects. A recent publication demonstrated the application of this entropy-based principle to the separation of various binary mixtures such as *n*-alkanes and *n*-alcohols in zeolites and MOFs,^60^ where the concentration of the species with smaller molar volume in the adsorbed phase increases as pore saturation is approached.

Motivated by these observations, we investigated the coadsorption of equimolar L2/D4, L4/D4, and L4/D5 mixtures in FOTNIN at 435 K. Although it would be best to select a nominal operating temperature based on an accurate equation of state for siloxane mixtures, this information is not currently available. For this reason, we adopted an operating temperature used previously for separating hexane isomers.^20^ L2/D4 was chosen as these species make up most of the total siloxane contaminants in biogas,^2−4^ with a difference in molar volume of 1 × 10^–4^ m^3^/mol at 435 K and 1 bar and L2 having the smaller molar volume. L4/D5 were chosen due to their small differences in molar volumes; the molar volume differences at 435 K and 1 bar in L4/D4 and L4/D5 are 6–8 × 10^–5^ m^3^/mol, respectively.

Configurational bias Monte Carlo (CBMC) simulations were performed to calculate the single-component isotherms for each linear siloxane (L2, L3, and L4), while continuous fractional component Monte Carlo (CFCMC) simulations were performed to calculate the single-component isotherms for each cyclic siloxane (D4, D5, D6) in FOTNIN at *T* = 435 K. We first did a convergence test at *T* = 435 K and *p* = 10,000 Pa with 10,000 cycles for equilibration and 100,000, 200,000, 300,000, and 400,000 cycles for production and found that 400,000 is required for sufficient accuracy (see Figure S8a). CFCMC simulations were performed to calculate the equimolar L2/D4 binary mixture isotherm at *T* = 435 K. For the equimolar L4/D4 and L4/D5 binary mixture isotherms, CFCMC/MD hybrid simulations were performed to increase the insertion acceptance ratios.^56^ Convergence tests showed that 1,000,000 cycles for production was required for L2/D4 and 200,000 cycles for production was sufficient for L4/D4 and L4/D5 (see Figure S8b,c).

CBMC and CFCMC simulations of the single-component adsorption isotherms of L2, L3, L4, D4, D5, and D6 in FOTNIN at *T* = 435 K are presented in Figure 4. The hierarchy of adsorption strengths for L2, L3, and L4 and D4, D5, and D6 is as expected; adsorption strength increases with chain length for linear siloxanes or ring size for cyclic siloxanes. At pressures similar to or higher than the vapor pressure of the bulk fluid phase, where the adsorbent is in contact with siloxane fluids in a liquid-like or liquid phase, the pores of the adsorbent are saturated with guest molecules. The saturation capacities of the siloxanes decrease with an increasing liquid molar volume. We estimated the saturated loading of each siloxane in FOTNIN by combining the accessible volume of FOTNIN, 0.00383 m^3^/kg_MOF_,^14^ with the siloxane’s bulk-phase liquid density^34^ (see Table S6). Saturation loadings estimated by using this approach are represented by dashed lines in Figure 4. Compared to the saturated loadings obtained from CBMC or CFCMC simulations, this method overestimated the saturated loading for all siloxanes. These discrepancies suggest the need for a molecular-size-dependent pore volume in order to more accurately estimate the saturation loadings. Tang et al. previously suggested using a probe-size-dependent scaling factor and a molecular-size-dependent pore volume to predict the saturation loadings of a wide range of adsorbates in MOFs.^59^ The scaling factor suggested by Tang et al. cannot be extrapolated to adsorbates with molecular weights as large as the siloxanes that we studied, but this concept suggests a path forward to make more reliable predictions of saturation loadings for siloxanes in the future. Adsorption occurs at higher pressures at 435 K (ca. 10^2^–10^4^ Pa) compared to adsorptions discussed in the previous section at 303 K (∼10 Pa) because pure fluid vapor pressures increase drastically from 303 to 435 K (see Figure S9).

To present results for mixture adsorption, we first discuss the coadsorption of L2 and D4 in FOTNIN. Figure 5a shows the CFCMC simulation data for the adsorption of an equimolar L2/D4 binary mixture at 435 K. At pressures below 10^5^ Pa, the bulk fluid mixture is a vapor. Selectivity at pressures below 10^5^ Pa favors D4 adsorption over L2, which is the component with larger molecular weight and greater binding strength. Selectivity reversal occurs at pressures above 10^5^ Pa, where the smaller species, L2, is preferentially adsorbed due to a higher packing efficiency.

For the adsorption of L4/D4 mixtures, CFCMC/MD simulations show that D4 is almost completely excluded under pore saturation conditions (see Figure 5b), so the adsorbed phase is almost exclusively occupied by L4. Configurational entropy tends to favor the linear and flexible L4 siloxane because they pack more efficiently within the pore than cyclic D4 siloxane. CFCMC/MD simulations of L4–D5 mixture adsorption are presented in Figure 5c. For pressures above 10^3^ Pa, at pore saturation conditions, cyclic D5 siloxane is progressively excluded from the pores, and the adsorbed phase mixture becomes richer in L4 due to configurational entropy effects.

## 6 Conclusions

A new united-atom Lennard-Jones-type FF has been developed for cyclic and linear siloxanes by fitting LJ parameters to bulk vapor–liquid equilibrium curves of D4, D5, and D6. To the best of our knowledge, this is the first FF for these species that accurately reproduces this bulk phase behavior. Interaction energies between MOFs and siloxanes predicted with this FF combined with the Universal Force Field (UFF) for MOF atoms show good agreement with dispersion-corrected DFT calculations and show considerably greater accuracy by this measure than the only previous FF for siloxane/MOF interactions. We performed tests comparing FF and dispersion-corrected DFT calculations in 55 different MOFs without open metal sites, giving good evidence of the transferability of our FF. D4 adsorption isotherms in FOTNIN and MIL-101 predicted with CFCMC simulations using this FF agree well with the limited experimental data that is available. Thus, our FF makes it possible for the first time to make quantitative predictions about both the bulk phase behavior of linear and cyclic siloxanes and the adsorption of these molecules in a broad class of MOFs.

We used our new FF to study mixture adsorption in the separation of binary mixtures of linear and cyclic siloxanes at high pore occupancies in a representative large-pore MOF, FOTNIN. At liquid-like densities in the adsorbed phase entropy effects play an important role, giving preferential adsorption for species that pack more efficiently in the MOF’s pores. No previous information from experiment or simulation was available for mixture adsorption of siloxanes, so our results indicate how simulations with accurate FFs can be a useful tool to understand the properties of these mixtures.

We note that all simulations in this work were performed in rigid structures, and all of the MOFs we considered except MIL-101 do not have open metal sites. It may be useful in the future to understand the specific interactions that are possible between siloxanes and open metal sites, as well as possible catalytic reactions at these sites, using DFT calculations. For all MOFs, making truly quantitative predictions of adsorption may require incorporation of framework flexibility effects, including effects associated with thermal vibrations of framework atoms and adsorbate-induced effects such as pore swelling, and simulation methods to account for these effects are available.^61,62^ For materials that are found to be of high interest for siloxane capture or siloxane separations, it may be important to assess the impact of these effects, especially the possible role of pore swelling in cases where adsorption reaches liquid-like densities.

## Supporting Information Available

Additional data and computational results: vapor–liquid coexistence curves (VLCC) for D4 using existing force fields, comparison of Coulombic interaction between D4–D4 dimer to FSAPT-D3MBJ interaction energy, intramolecular potential parameters for cyclic and linear siloxanes, comparison of VLCC for methane computed using MD and GEMC, characteristics of 55 non-open metal site MOFs from the Core MOF database, convergence test for CBMC and CFCMC simulations, molar volumes of selected siloxanes from the NIST Webbook, vapor pressure curves of selected siloxanes from the NIST Webbook. Complete collection of data for VLCC of cyclic and linear siloxanes from MD simulations; adsorption isotherms of D4 in FOTNIN and MIL-101 from CFCMC simulations; single-component adsorption isotherms data of L2, L3, L4, D4, D5, and D6 in FOTNIN from CFCMC and CBMC simulations; and binary adsorption isotherms data of L2/D4, L4/D4, and L4/D5 in FOTNIN from CFCMC and CFCMC/MD hybrid simulations (PDF)
Chng et al.SI Data (ZIP)
