# Supplementary material for: Quantitative Simulations of Siloxane Adsorption in Metal–Organic Frameworks
Source: ACS Appl Mater Interfaces. 2023 Jul 26;15(31):37828–36. doi: 10.1021/acsami.3c07158 (PMC10416143; doi:10.1021/acsami.3c07158)
Supplement: Supplementary file 1 — am3c07158_si_001.pdf [file am3c07158_si_001.pdf]

Supporting Information:
Quantitative simulations of siloxane adsorption in metal-organic frameworks

Jia Yuan Chng† and David S. Sholl*,†,‡

†School of Chemical & Biomolecular Engineering, Georgia Institute of Technology, Atlanta, Georgia 30332-0100, United States.

‡Oak Ridge National Laboratory, Oak Ridge, Tennessee 37830, United States.

E-mail: shollds@ornl.gov

The attached Excel spreadsheet contains data for:

1. MD simulations data for vapor-liquid coexistence curves for cyclic and linear siloxanes (Figure 1).
2. PBE-D3 DFT, new FF and UFF data for D4-MOFs binding energies (Figure 2).
3. CFCMC simulations data for adsorption isotherms of D4 in FOTNIN and MIL-101 at 303 K (Figure 3).
4. CBMC simulation data for single component adsorption isotherms of L2, L3 and L4, and CFCMC simulation data for single component adsorption isotherms of D4, D5 and D6 in FOTNIN at 435 K (Figure 4).
5. CFCMC simulation data for equimolar binary L2/D4 mixture adsorption in FOTNIN at 435 K (Figure 5a).
6. CFCMC/MD simulation data for equimolar binary L4/D4 and L4/D5 mixture adsorption in FOTNIN at 435 K (Figures 5b and 5c).

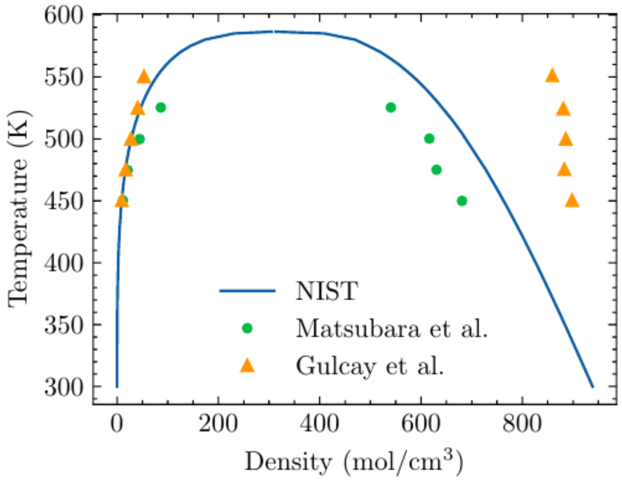

Figure S1: Vapor-liquid coexistence curves for D4 computed using the FFs of Gulcay et al.[1] and Matsubara et al.[2] compared to experimental data as reported by NIST (solid curve).

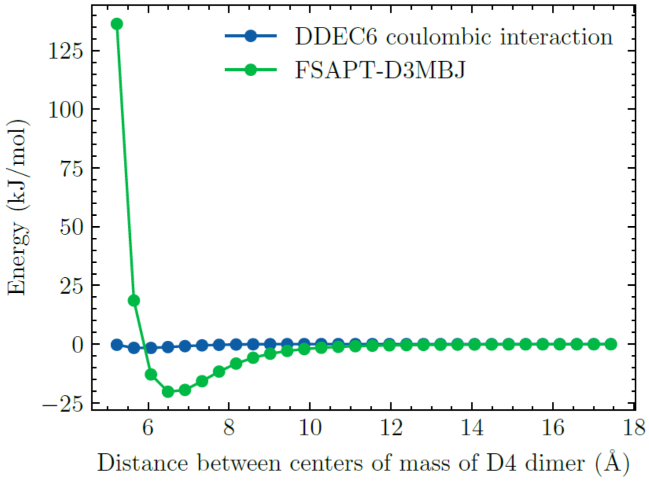

Figure S2: D4–D4 dimer overall interaction energy at the F-SAPT0-D3M(BJ) level and Coulombic interaction energy plotted as a function of distance between molecules forming the dimer. The relative orientations of the D4–D4 dimer were fixed as shown in Figure S3.

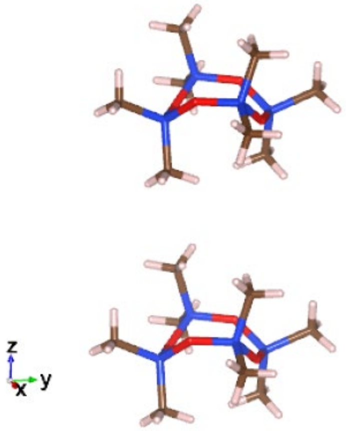

Figure S3: Relative orientations of D4–D4 dimer in the calculations used for Fig. S2.

Table S1: Equilibrium bond lengths for siloxanes.

**Angle**	**Length** (Å)
**Si-O**	1.658
**O-CH₃**	1.873

Table S2: Equilibrium bond angles and force constants for cyclic and linear siloxanes. Subscripts "c" and "l" denote cyclic and linear respectively.

**Angle**	$\boldsymbol{\theta_0}$(°)	$\boldsymbol{k_\theta}$ (kcal/mol)
**(O-Si-O)$_c$**	109.870	185.206
**(Si-O-Si)$_c$**	149.520	120.026
**(CH_3-Si-CH_3)$_c$**	113.070	124.199
**(CH_3–Si–O)$_c$**	108.760	99.956
**(O–Si–O)$_l$**	108.525	94.500
**(Si–O–Si)$_l$**	144.502	14.140
**(CH_3–Si–CH_3)$_l$**	111.022	49.970
**(CH_3–Si–O)$_l$**	108.830	49.970

Table S3: Torsional parameters for cyclic and linear siloxanes. Subscripts “c” and “l” denote cyclic and linear respectively.

Dihedral type	c_0 (kcal/mol)	c_1 (kcal/mol)	c_2 (kcal/mol)	c_3 (kcal/mol)
**$(Si–O–Si–CH_3)_c$**	2.2253	1.4414	0.3254	1.1093
**$(Si–O–Si–O)_c$**	23.291	35.209	17.644	3.1477
	k_{tors} **(kcal/mol)**	n		
**$(Si–O–Si–CH_3)_l$**	0.01	3		
**$(Si–O–Si–O)_l$**	0.225	1		

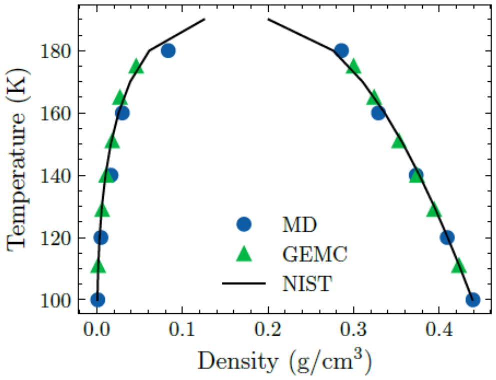

Figure S4: Comparison of vapor-liquid equilibrium (VLE) curve for methane for the TraPPE force field calculated using molecular dynamics (MD) and from Gibbs ensemble Monte Carlo (GEMC).[3]

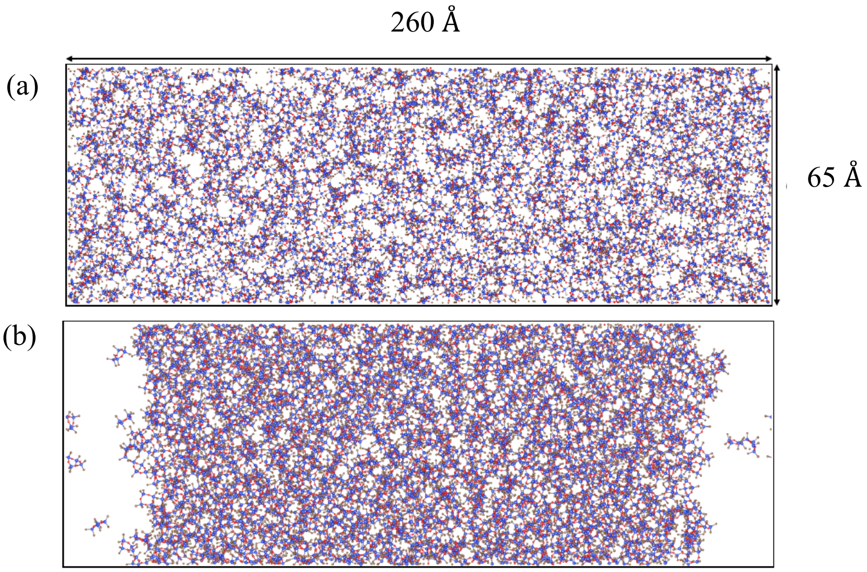

Figure S5: Snapshots of configurations for D4 before and after temperature quenching: (a) Snapshot of high temperature configuration for D4 ($T > T_{critical}$), (b) Snapshot of final configuration for D4 ($T = T_{desired}$).

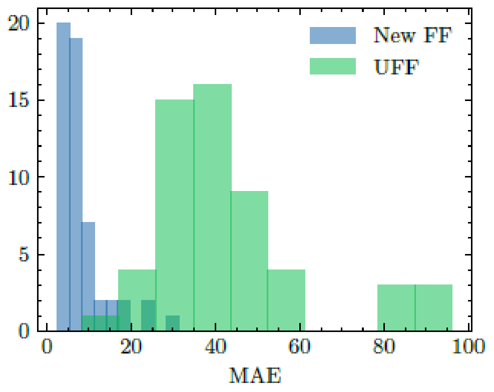

Figure S6: Mean absolute error (MAE) of the interaction energies of D4 in 55 MOFs at the PBE-D3 level and our new FF (blue) and UFF used in the work by Gulcay et al.[1] (green).

Table S4: Characteristics of the 55 non-open metal site MOFs from the Core MOF database.[4]

MOF	LCD	Void fraction	MOF	LCD	Void fraction
FOTNIN	33.62	0.92	BOLZIN	18.71	0.81
CUSYAR	28.01	0.91	MEJMOE	13.36	0.81
RUTNOK	24.61	0.9	ICITEU	19.8	0.81
DAJWET	28.06	0.88	LAWGOG	15.08	0.8
HOHMEX	18.78	0.88	LAWGUM	15.1	0.8
ECOKAJ	19	0.87	LAWGEW	15.06	0.8
ADATAC	26.34	0.87	KARNAU	15.53	0.8
WUHDAG	25.24	0.87	LAWGIA	15.07	0.8
XUKYEI	13.17	0.87	PUZLUS	15.09	0.8
RUBDUP	21.1	0.87	VUSKEA	15	0.8
WUHCUZ	23.13	0.86	VUSKAW	14.99	0.8
EDUVOO	20.93	0.86	VAZTOG	15.08	0.8
HOMXIR	23.7	0.84	NEYVEU	20.18	0.8
SUGDIJ	13	0.84	HAFTOZ	15.37	0.78
ALULEZ	18.76	0.83	FEFDEB	13.11	0.77
BIBXOB	19.69	0.83	EDUSUR	14.97	0.76
ICAQOU	20.3	0.82	OXOLAP	19.1	0.76
GISNED	16.88	0.82	YURJUR	13.69	0.76
IYOWID	20.52	0.82	KULMEK	13.21	0.75
CAVPEW	20.26	0.82	ABEXEN	18.81	0.75
CAVPIA	19.92	0.81	HIFTOG	13.28	0.75
MUBZOA	18.73	0.81	WUTBEU	12.66	0.75
LAWFOF	15.1	0.81	GUPCAW	13.65	0.74
LAWFUL	15.1	0.81	YUXQIS	17.65	0.74
LAWGAS	15.09	0.81	BIBXIV	18.49	0.73
PEDRIA	15.09	0.81	BINCUY	13.31	0.72
EDUSIF	15.05	0.81	GITVEL	15.25	0.72
NEXVET	15.05	0.81			

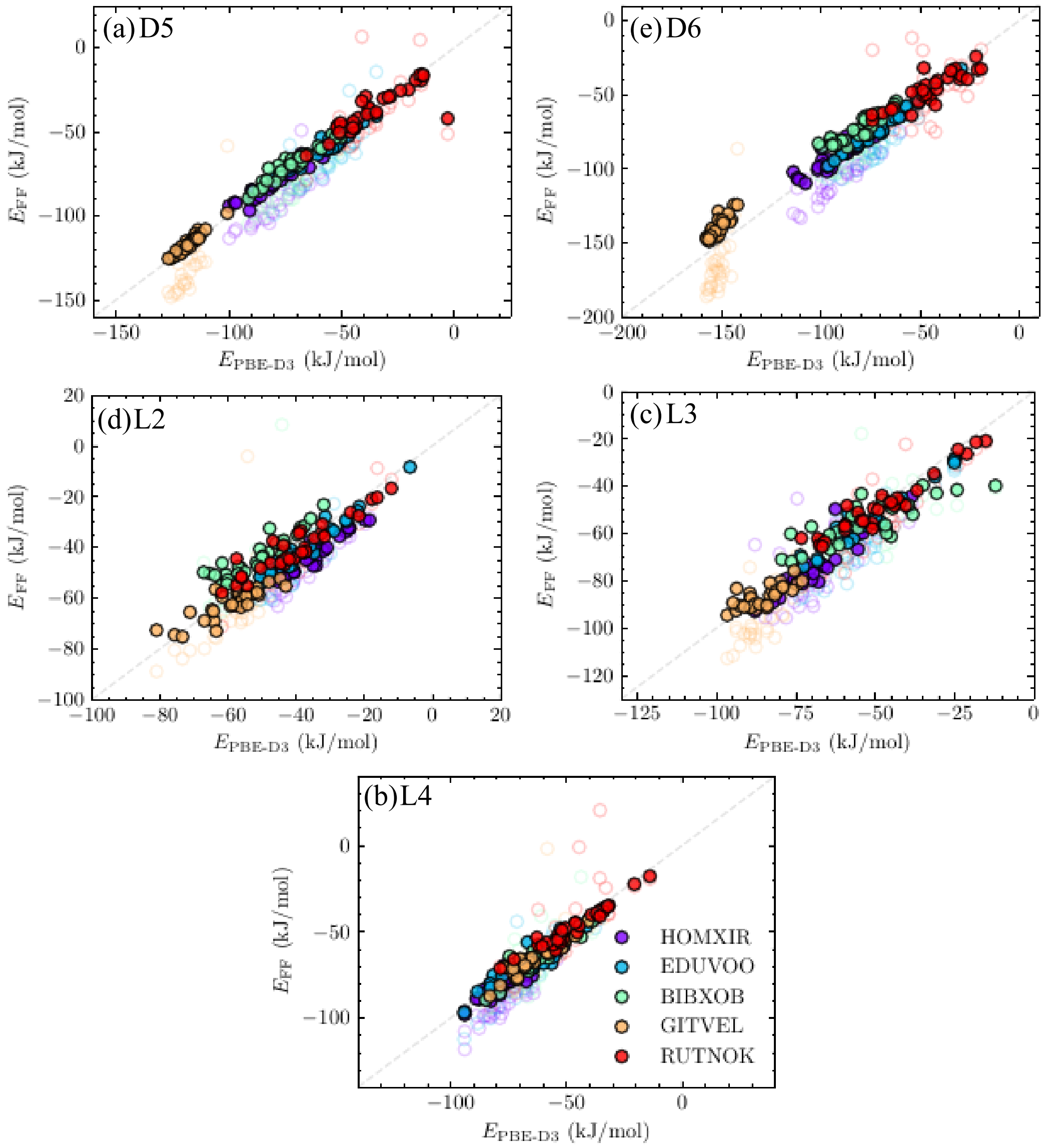

Figure S7: Comparison of the interaction energies of (a) D5, (b) D6, (c) L2, (d) L3 and (e) L4 in 5 randomly selected MOFs from the list of 55 MOFs at the PBE-D3 level and our new FF (filled circles) and Gulcay et al.'s FF[1] (unfilled circles), with data from 30 independent configurations in each MOF. MOFs are represented by different colors. Mean absolute error (MAE) of the binding energies are tabulated in Table S5.

Table S5: Mean absolute error (MAE) of the binding energies of D5, D6, L2, L3 and L4 in 5 randomly selected MOFs at the PBE-D3 level and our new FF and UFF used in the work by Gulcay et al.[1]

Molecule	New FF (kJ/mol)	UFF (kJ/mol)
D5	4	14
D6	6	15
L2	5	9
L3	4	12
L4	4	13

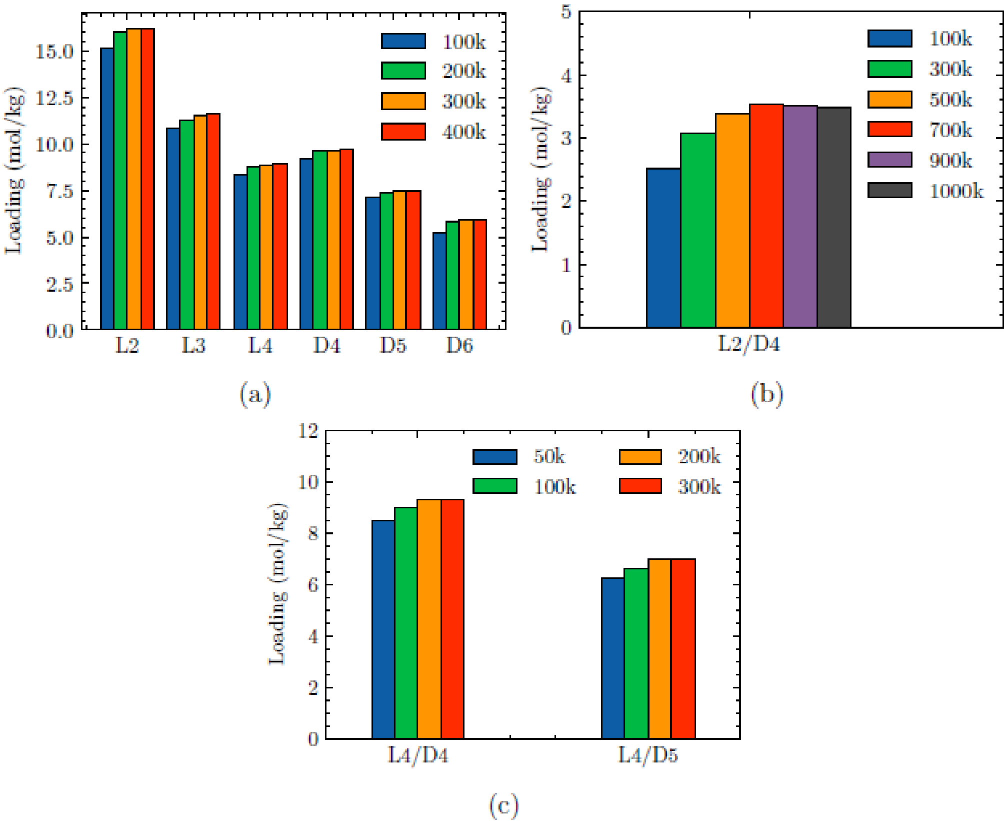

Figure S8: Convergence tests for simulations of single component and binary equimolar mixture adsorption isotherms in FOTNIN: (a) Convergence tests for single component configurational bias Monte Carlo (CBMC) simulations for L2, L3 and L4, and continuous fractional component Monte Carlo (CFCMC) simulations for D4, D5 and D6 in FOTNIN at T = 435 K and p = 10,000 Pa with 100,000, 200,000, 300,000, and 400,000 cycles for production. Each vertical bar shows the resulting loading of siloxanes in FOTNIN as a function of number of production cycles. (b) Convergence tests for CFCMC simulations for an equimolar L2/D4 binary mixture in FOTNIN at T = 435 K and p = 10,000 Pa with 100,000, 300,000, 500,000, 700,000, 900,000 and 1,000,000 cycles for production. Each vertical bar shows the resulting D4 loading of D4 as a function of the number of production cycles. (c) Convergence tests for CFCMC/MD hybrid simulations for equimolar L4/D4 and L4/D5 binary mixtures in FOTNIN at T = 435 K and p = 10,000 Pa with 50,000, 100,000, 200,000 and 300,000 cycles for production. Each vertical bar shows the resulting loading of L4 as a function of the number of production cycles.

Table S6: Molar volume of siloxanes.[5]

Molecule	Molar volume at T=303 K, P = 1 bar (m^3/mol)	Molar volume at T=435 K, P = 1 bar (m^3/mol)	Molar volume at T=435 K, P = 10 bar (m^3/mol)
L2	0.000214	0.034896	0.000273
L3	0.000291	0.033743	0.000354
L4	0.000367	0.000440	0.000437
D4	0.000313	0.000378	0.000376
D5	0.000390	0.000461	0.000459
D6	0.000460	0.000547	0.000543

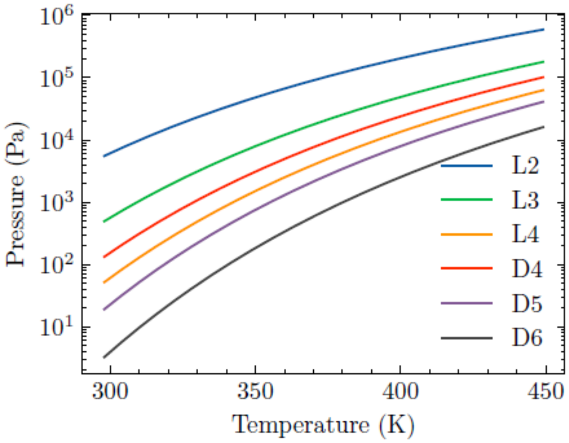

Figure S9: Vapor pressure of selected siloxanes using data from the NIST Webbook.[5]

References

(1) Gulcay, E.; Iacomi, P.; Ko, Y.; Chang, J.-S.; Rioland, G.; Devautour-Vinot, S.; Maurin, G. Breaking the Upper Bound of Siloxane Uptake: Metal–Organic Frameworks as an Adsorbent Platform. J. Mater. Chem. A 2021, 9, 12711–12720.

(2) Matsubara, H.; Pichierri, F.; Kurihara, K. Design of a Versatile Force Field for the Large-Scale Molecular Simulation of Solid and Liquid OMCTS. Journal of Chemical Theory and Computation 2010, 6, 1334–1340.

(3) Martin, M. G.; Siepmann, J. I. Transferable Potentials for Phase Equilibria. 1. United- Atom Description of n-Alkanes. The Journal of Physical Chemistry B 1998, 102, 2569–2577.

(4) Chung, Y. G.; Haldoupis, E.; Bucior, B. J.; Haranczyk, M.; Lee, S.; Zhang, H.; Vogiatzis, K. D.; Milisavljevic, M.; Ling, S.; Camp, J. S.; Slater, B.; Siepmann, J. I.; Sholl, D. S.; Snurr, R. Q. Advances, Updates, and Analytics for the Computation-Ready, Experimental Metal–Organic Framework Database: CoRE MOF 2019. Journal of Chemical & Engineering Data 2019, 64, 5985–5998.

(5) Linstrom, P.; Mallard, W. NIST Chemistry WebBook, NIST Standard Reference Database Number 69. National Institute of Standards and Technology 2022.
